# Supplementary material for: Are carbon emissions trading and green financial instruments synergistic? -Comprehensive quantitative research based on content analysis
Source: PLoS One. 2024 Mar 7;19(3):e0298601. doi: 10.1371/journal.pone.0298601 (PMC10919601; doi:10.1371/journal.pone.0298601)
Supplement: S1 Data — (ZIP) [file pone.0298601.s001.zip › Data/地级市Z_F工作报告.zip!/╔╜╢1⁄2/╤╠╠¿╩╨/└┤╫╘╣1⁄2╓┌║┼í╛┬φ┐╦╩2╛▌═°í┐_2019.docx]

各位代表：

现在，我代表市人民政府向大会报告工作，请予审议，并请市政协委员和其他列席会议的同志提出意见。

一、2018年工作回顾

2018年是全面贯彻党的十九大精神的开局之年，是改革开放40周年，是烟台发展历程中很不平凡的一年。6月中旬，习近平总书记亲临烟台视察并作出重要指示，极大鼓舞和激励了全市人民的信心，为烟台发展指明了方向，提供了根本遵循和强大动力。我市被列入山东新旧动能转换综合试验区三个核心区之一，省委、省政府主要领导多次来我市调研指导工作，全力支持烟台高质量发展。中共烟台市委召开十三届五次全会，深入学习贯彻习近平总书记视察山东、视察烟台重要讲话精神，确立了“加快建设制造业强市、海洋经济大市、宜业宜居宜游城市和现代化国际滨海城市”的目标定位。一年来，全市上下在市委的坚强领导下，以习近平新时代中国特色社会主义思想为指导，全面落实新发展理念，加快新旧动能转换，着力推动高质量发展，全市经济稳中有进，社会和谐稳定，较好完成了年初确定的工作目标。预计，全年全市实现生产总值7900亿元，增长6.5%左右；一般公共预算收入636.6亿元，增长6%；规模以上工业增加值增长6.5%左右，固定资产投资增长7%左右，社会消费品零售总额增长8%左右，城镇居民人均可支配收入增长7.6%左右，农村居民人均可支配收入增长7.3%左右。

（一）新旧动能转换快速起步。加快淘汰落后产能，推动传统产业转型升级，大力培育新兴产业，“八大主导产业”不断发展壮大，新旧动能加快接续转换。推动制造业高质量发展。全面实施制造业强市战略，全年实现规模以上工业总产值1.04万亿元，增长7.1%，工业主营业务收入、利润保持全省领先。产业结构、能源结构持续优化，127个过亿元技改项目完成投资增长10%。战略性新兴产业产值占规上工业总产值比重达到24%，节能环保、生物技术、高端装备制造产业产值分别增长24.1%、22.3%、15.6%，医药健康产业总收入增长12%。万元生产总值能耗下降3.4%。骨干企业引领支撑作用进一步增强，万华二期乙烯项目开工建设，上汽通用东岳新车型投产，玲珑轮胎荣获中国工业大奖，台海材料科技、台海玛努尔核电设备、南山铝业入选国家工业强基工程，鲁花集团、恒邦集团、道恩集团上榜中国民营企业500强，绿叶制药、中节能万润股份成为国家技术创新示范企业，中集来福士建造的“蓝鲸1号”获中国优秀工业设计金奖，14家企业入选山东省首批百家“瞪羚”企业名单。创新能力持续提升。大力实施创新驱动战略，新培育高新技术企业83家，总数达到453家，新备案科技型中小企业856家，高新技术产业产值占规模以上工业总产值比重达到46.7%，高于全省平均水平10.1个百分点，全社会研发投入占生产总值的比重达到2.58%。新发展省级以上工程技术研究中心、企业技术中心、重点实验室37家，总数达到278家。获批国家首批知识产权军民融合试点。烟台校地合作示范基地引进建设了53个高端创新平台，中德国家级战略合作项目——中德工业设计中心落户烟台。22个项目获省级以上科技奖，3个项目获国家科技奖，2项专利获中国专利金奖，海军航空大学何友院士获山东省最高科学技术奖。大力推动民营经济发展。认真落实习近平总书记在民营企业座谈会上的重要讲话精神，出台支持非公有制经济健康发展等政策措施，落实减税降费政策，全力帮助民营企业解决发展中的困难，全力支持民营企业健康成长。389家企业完成规范化公司制改制，331家小微企业升级为规模以上企业。着力发展海洋经济。编制出台海洋强市建设规划，长岛海洋生态文明综合试验区建设稳步推进。海洋生产总值达到2230亿元，增长11%，海洋战略性新兴产业产值增长16.7%。海洋牧场发展迅速，创建7处国家级、16处省级海洋牧场示范区，海洋牧场突破100万亩，居全国首位。全市港口货物吞吐量达到4.4亿吨，烟台港位列全国沿海港口第8位。加快推进现代服务业发展。积极培育现代金融业，全市金融机构本外币存贷款余额分别达到8211亿元和5550亿元，实现直接融资468亿元，各类基金实缴规模519亿元。获批国家资本项目收入结汇支付便利化改革试点。全市旅游消费总额突破1000亿元。蓬莱国际机场旅客吞吐量达到843万人次。电子商务交易额增长30.2%。获批全国流通领域现代供应链体系试点。成功举办国际果蔬食品博览会、医药创新与发展国际会议、首届世界老年旅游大会等一批重要展会。

（二）改革开放活力持续增强。重点领域改革全面发力。新争取国家、省改革试点60项，研究出台改革方案100余项。扎实推进“一次办好”改革，开展优化营商环境10个专项行动，取消和调整行政权力事项852项，组建成立市县两级行政审批服务局，推行政务服务“一窗受理”“一链办理”“一网通办”，实现了“一枚印章管审批”，“多证合一”扩大到“45证合一”，企业开办、项目投资、不动产登记等审批时间大幅缩短。深化企业改革，国有企业“三供一业”分离移交工作全面完成，市属经营性国有资产实现统一监管，市管企业营业收入增长10%。深化园区改革，烟台经济技术开发区等4个国家级园区探索实施大部门制、全员聘用制、绩效薪酬制，烟台高新区扩大管辖范围，烟台保税港区启动西迁置换。市区规划建设管理全面实现市级统筹。市属公办高职院校及市区部分高中段学校实现市级统筹管理。84.6%的涉农村居完成集体产权制度改革。对外开放取得丰硕成果。大力实施“双招双引”，整合市县两级内外资招商职能，积极参加山东儒商大会、香港（山东）周等活动，成功举办“乡情·乡约烟台之夜”等活动，引进世界500强、中国500强、民营500强企业投资项目75个，全年到位市外资金1050亿元，实际使用外资176亿元，增长21.5%。加大招才引智力度，在人才落户、子女入园入学等方面出台系列政策，全年引进各类人才3.7万人，其中院士20人、“千人计划”专家52人，分别增长30%和60%，5人入选国家“千人计划”企业创新长期项目，占全省一半以上。中韩（烟台）产业园挂牌成立，全市实际使用韩资增长31.5%。持续优化外贸发展环境，口岸整体通关时间压缩1/3，开通烟台至阿拉木图中欧班列，获批省级服务外包示范城市和3个国家级外贸转型升级示范基地，完成进出口3080亿元，外贸实现平稳发展。

（三）乡村振兴开局良好。出台乡村振兴战略规划，构建乡村振兴发展新格局。农业产业化水平进一步提高。粮食生产喜获丰收，全年粮食总产36.7亿斤。新增省级农业产业化龙头企业18家、农民合作社1370家、家庭农场592家，节水灌溉22.2万亩、水肥一体化26.3万亩，国家级现代农业产业园“栖霞模式”在全国推广。新认证“三品一标”农产品131个。畜禽标准化规模养殖比重达到87%以上。主要农作物综合机械化水平达到91%。推进耕地数量、质量、生态“三位一体”保护，连年实现耕地总量动态平衡。农村生产生活条件进一步改善。大力实施农村环境综合整治，创建省级美丽乡村示范村42个，建设“四好农村路”，改造农村危房1451户、农厕5.1万户、清洁取暖3.1万户，建制镇生活污水集中处理率提高到86.5%，解决了239个村饮用水水质安全问题。完成99处乡镇卫生院标准化建设，创建22个省级卫生乡镇。16个小城镇入选全国千强镇。乡村文明程度进一步提升。大力推动乡村文化振兴，创建省级“乡村文明家园”28个，建成县级新时代文明实践中心15个、乡镇分中心68个、村居实践站1133个。农村治理能力进一步增强。顺利完成村（居）委会换届选举。2814个村成立新型农村集体经济组织。提升完善1143个农村社区服务中心，共建共治共享的城乡社区治理体系进一步完善。

（四）三大攻坚战扎实推进。重大风险得到有效防控。建立“政监银企”会商机制，积极化解企业流动性、担保圈等重点领域风险，担保圈个数和涉及企业数分别减少4个和295户。重拳打击逃废银行债务行为，有效遏制非法集资，刑事立案70起，涉及金额22.9亿元。银行机构不良贷款率低于全省平均水平。顺利完成存量政府债务置换工作。脱贫攻坚任务基本完成。高度重视精准扶贫、精准脱贫，全年安排财政扶贫专项资金1.4亿元，发放富民生产贷2.18亿元，实施662个产业扶贫项目，全市65050名建档立卡贫困人口基本实现稳定脱贫。援藏援疆和扶贫协作工作取得实效。生态文明建设力度空前。牢固树立“绿水青山就是金山银山”理念，以坚定的决心全面整改中央、省生态环保督察反馈问题，办结群众信访案件1827件。大力实施大气、水、土壤污染综合防治，关闭淘汰化工企业152家，关停取缔和整改提升“散乱污”企业6012家，市区空气质量达到国家二级标准。自然保护区内关停采矿探矿点102处、拆除风电机组236台，全力清理核心区、缓冲区内养殖项目和违建项目。全面落实河长制湖长制，河库水系连通、病险水库加固等75项全域治水工程顺利推进，黑臭水体整治通过国家验收，集中式饮用水源地水质稳定达标，成为全国水生态文明城市。完成造林和生态修复11.7万亩、森林抚育20万亩、湿地保护与修复8.4万亩。

（五）城市功能品质进一步提高。坚持以新发展理念统筹城市规划，编制城市发展战略研究等各类规划72项，全域规划体系基本建成。大力推进重大基础设施建设，海阳核电1号、2号机组投入商运，龙烟铁路、龙青高速公路建成通车，大莱龙铁路扩能改造工程、文莱高速公路开工建设，环渤海潍烟高铁开展前期工作。实施163项市区城建重点项目，完成投资258亿元。新建整修市区道路23条，打通断头路23条，滨海西路夹河桥通车运行。全面开展城市环境综合治理，开工建设棚改安置房6.17万套，改造老旧小区243个、背街小巷307条，拆除违章建筑122万平方米，全市新增天然气用户8.3万户，新增集中供热面积1081万平方米。完成29平方公里海绵城市建设和排水系统雨污分流改造。套子湾再生水项目一期工程试运行。我市入选全国首批历史建筑保护利用试点城市，荣获山东省适宜人居环境奖。

（六）民生保障水平不断提升。坚持以人为本、民生优先，千方百计保障和改善民生。新增城镇就业连续14年超过13万人，城镇登记失业率3.06%。高度重视保障困难群众基本生活，县均城乡低保标准分别提高到每人每月590元和411元，救助困难群众130万人次，筹集共有产权住房1284套，发放住房租赁补贴1.6万户。全面推开职工长期护理保险制度，启动居民长期护理保险试点。坚持优先发展教育，投入16.8亿元实施义务教育学校“全面改薄”、消除大班额和高中阶段教育普及攻坚，新建改建学校45所、幼儿园107所，办学条件进一步改善。全面推进国家健康城市和医养结合试点市建设，建成骨科医学中心和中医药预防保健中心，通过国家卫生城市复审。新建47处医养结合服务机构，新增床位10470张。群众性文体活动丰富多彩，开展文化惠民演出2400场，建成13处城市书房，举办全民阅读活动3000多场。新建316处体育场所，烟台健儿在省运会上获得金牌总数第三名。牢固树立红线意识和底线思维，深入开展“大快严”集中行动，安全生产形势稳定向好。食品安全综合指数居全国示范城市首位。深入开展扫黑除恶专项斗争，侦办黑社会性质犯罪组织7个、恶势力犯罪集团31个、涉恶类团伙313个，广大群众拍手称快。严厉打击各类违法犯罪活动，刑事立案同比下降31.5%，反电信诈骗、疑难逃犯攻坚会战工作成果居全省首位。双拥共建、国防动员、民兵预备役、人民防空工作顺利开展，积极落实退役军人相关政策措施。国家安全、司法、信访稳定、审计、统计、史志、档案、仲裁、机关事务、新闻出版、广播电视、民族宗教和气象、地震、打私等工作取得新成绩，人口计生、老龄、妇女、儿童、残疾人、红十字事业健康发展。

（七）政府自身建设全面加强。认真学习贯彻习近平新时代中国特色社会主义思想和党的十九大精神，树牢“四个意识”，坚定“四个自信”，做到“两个维护”，坚定不移贯彻落实上级各项决策部署，以高度的政治自觉抓好中央巡视反馈问题整改，严格落实意识形态工作责任制，全面提升政府系统党建水平和执行力。自觉接受市人大及其常委会监督、政协民主监督、社会舆论监督，认真听取社会各界意见，207件人大代表建议、453件政协提案全部办结。有序推进政府机构改革，坚持依法行政，提升政务公开规范化水平，设立市级统一的政务信息资源共享交换平台，整合全市“12345”政务服务热线。完成市公共信用信息平台一期建设，全面建立失信联合惩戒机制，城市信用综合指数居全国地级市第2位。深入开展“大学习、大调研、大改进”，驰而不息纠治“四风”，坚持不懈反腐倡廉，政务生态环境持续改善。

各位代表，2018年是烟台发展历程中具有里程碑意义的重要一年。这一年，我们牢记总书记嘱托，在市委的坚强领导下，积极开展“思想大解放、作风大改进、工作大落实”活动，深入查找在思想、作风、工作等方面的问题和不足，瞄准“走在前列”不动摇，主动对标先进，树立在14个沿海开放城市中位次前移、在省内“保三争二抢第一”工作导向，以坚定的步伐走在全省前列。这一年，我们聚焦“三重”工作集中用力，扎实推进80项重点工程、重点项目、重大事项，强基础、补短板、闯新路、增动能，以点带面推动各项工作扎实开展。这一年，我们面对经济下行压力，坚定信心、迎难而上，加强经济运行调度，为企业解难纾困，出台一系列实打实的政策措施，积极推动国有企业改革发展，大力支持民营经济健康发展，为企业发放信贷周转金、兑现奖补资金、减税降费331亿元。这一年，我们始终坚持以人民为中心的发展思想，民生支出达到585.8亿元，扎实办好民生实事，着力补齐民生短板，优化公共服务供给，进一步增强了广大群众的获得感、幸福感和安全感。这一年，我们认真总结改革开放40年发展经验，打出了全面深化改革的组合拳，拉开了“双招双引”序幕，有力激发了发展动能，增强了发展活力。全国工业类“双百企业”现场交流会在烟台召开，向全国推广万华集团改革创新经验。全国海洋牧场建设工作现场会观摩了我市海工装备制造基地和海洋牧场示范区建设，烟台“海工+牧场”模式走在全国前列。

各位代表，过去一年的成绩来之不易，这是以习近平同志为核心的党中央亲切关怀的结果，是省委、省政府正确领导的结果，是市委带领全市人民团结一心、努力奋斗的结果，是市人大、市政协和各民主党派、工商联、无党派人士以及社会各界监督支持的结果。在此，我代表市人民政府，向全市人民、各界人士，向驻烟部队、武警官兵和公安干警，向中央省属驻烟单位，向老领导、老同志，向所有关心支持烟台发展的海内外朋友们，表示衷心的感谢并致以崇高的敬意！

在肯定成绩的同时，我们也清醒地看到，发展中还存在不少困难和不足，主要是：外部环境错综复杂，经济下行压力持续加大，部分指标低于预期目标。大项目好项目储备不足，创新能力有待进一步增强，新旧动能需要加快转换。改革进展尚不够平衡，一些重要领域改革事项仍推进较慢。开放型经济结构需持续优化，外经贸环境不稳定不确定因素增多。城乡基础设施建设存在不少短板，交通、能源、水源等瓶颈制约仍然比较突出。民生领域困难问题较多，上学、看病、出行等很多群众关心的热点难点问题有待解决。营商环境还有很多方面不尽人意，政府职能转变和制度创新仍有不足，一些干部思想作风、能力素质、担当精神还不能完全适应新时代发展要求，一些领域腐败问题多发频发。对上述问题，我们一定高度重视，努力加以解决。

二、2019年工作安排

2019年是新中国成立70周年，是全面建成小康社会关键之年，我们面临着新一轮改革开放机遇、新技术革命机遇、实体经济大发展机遇、绿色发展机遇和新旧动能转换等大好机遇，做好今年的工作意义重大。

2019年政府工作的总体思路是：以习近平新时代中国特色社会主义思想为指导，认真贯彻党的十九大和十九届二中、三中全会精神，深入落实习近平总书记视察山东、视察烟台重要讲话精神，统筹推进“五位一体”总体布局，协调推进“四个全面”战略布局，以走在前列为目标，坚持稳中求进工作总基调，坚持新发展理念，坚持推动高质量发展，坚持以供给侧结构性改革为主线，坚持深化市场化改革、扩大高水平开放，加快实施新旧动能转换重大工程，加快推进100项“三重”工作，加快办好100项惠民实事，更有成效地稳增长、促改革、调结构、惠民生、防风险，更加坚定地稳就业、稳金融、稳外贸、稳外资、稳投资、稳预期，促进经济持续健康发展和社会和谐稳定，奋力建设制造业强市、海洋经济大市、宜业宜居宜游城市和现代化国际滨海城市，以优异成绩庆祝中华人民共和国成立70周年。

今年全市经济社会发展的主要预期目标是：生产总值增长6.5%左右，一般公共预算收入增长5.5%左右，规模以上工业增加值增长6.5%左右，固定资产投资增长7%左右，社会消费品零售总额增长7.5%左右，城镇和农村居民人均可支配收入均增长7%以上，外贸进出口、实际使用外资保持稳定增长，居民消费价格涨幅3%左右，全面完成上级下达的节能减排约束性指标。

上述预期目标，既统筹考虑了全面建成小康社会需要，也充分估计了当前复杂的经济形势，既体现了稳中求进工作总基调，保持经济运行在合理区间，又为产业转型升级和新旧动能转换留出空间，有利于有效应对风险挑战，推动高质量发展行稳致远。

今年的政府工作，突出抓好以下九个方面：

（一）全力培强主导产业，筑牢高质量发展根基。抓好总投资3274.5亿元的161个市级重点项目，培育壮大“八大主导产业”，构建更具竞争力的现代产业体系。现代海洋产业，巩固海工装备产业优势，以中集来福士为依托，积极参与组建中国海工集团，争取中国海工北方中心落户我市，加快研发制造新型远洋渔船装备，建设全国重要的海工装备之城。装备制造产业，重点推进上汽通用CVT变速器、南山高端铝材等项目，加快布局新能源汽车、通用航空等战略性新兴产业，推进重点项目加快落地，建设国内一流的高端装备基地。电子信息产业，着眼新一代信息技术产业发展趋势，推动信息化与制造业深度融合，加快布局大数据、人工智能、虚拟现实等前沿产业，重点推进富士康半导体等项目，建设国内重要的消费类电子产品研发制造基地。高端化工产业，加紧推进万华二期乙烯项目建设，争取早日建成投产。积极参与山东地炼重组，争取万华裕龙炼化新材料项目尽快开工建设，以重大项目增创发展新优势。医养健康产业，建设医药研发公共服务平台，推进东方海洋精准医疗科技园、业达国际生物医药创新孵化中心等项目，打造国际生命科学创新示范区。高效农业，加快智慧农业发展，新认定市级以上现代农业产业园和田园综合体8个，新发展家庭农场160家、农民专业合作社500家，积极发展特色小镇、康养基地、“电商村”等新业态。文化旅游产业，叫响“仙境海岸·鲜美烟台”城市旅游形象品牌。围绕避暑休闲、滨海度假、文化观光、乡村体验，统筹养马岛、芝罘岛、崆峒岛等海岛旅游，塑造葡萄酒文化旅游等特色旅游品牌，加快实施一批重要文旅项目建设，争创长岛国家级旅游度假区，推动我市旅游由观光型向休闲度假型转变。现代物流产业，培育发展现代供应链物流，推进宝能烟台国际物流中心、传化智慧物流科技城、龙口港智能仓储中心等项目建设，新增星级物流企业（园区）2家，社会物流总额位居全省前列。

（二）实施创新驱动发展战略，加快推进新旧动能转换。全面落实“巩固、增强、提升、畅通”八字方针，坚持把创新作为第一动力，深入推进济青烟国家科技成果转移转化示范区建设，推动经济发展质量变革、效率变革、动力变革。全面激发创新活力。大力促进“大众创业、万众创新”，坚持以“四新”促“四化”， 积极实施“现代优势产业集群+人工智能”行动，依托物联网、云计算等技术，促进产业升级换代和智慧化、数字化改造提升，推动产业加速向智能化、绿色化迈进。重视科技服务业发展，支持创客空间、各类孵化器建设。推动中德工业设计中心、烟台核电研发中心、中科院育成中心、中科（烟台）大数据研究院、海尔市场创新产业园等一批重要创新平台建设。重视支持驻烟高校发展，发挥好高校院所的智库作用，加强校地合作示范基地建设，切实增强城市创新能力、激发创造活力。发挥企业创新主体作用。支持企业加大研发投入，走“专精特新”发展道路，集中实施15个重大科技创新工程项目，大力培育“独角兽”企业、“瞪羚”企业和制造业单项冠军，新增高新技术企业150家、科技型中小企业300家。加强知识产权保护运用，有效发明专利达到7000件以上。坚定不移支持民营经济发展。民营经济是推动新旧动能转换的重要力量。要坚持“两个毫不动摇”，完善领导干部常态化联系服务企业制度，全面落实支持民营经济高质量发展的政策措施，加大人才、金融、创新等方面支持力度，进一步降低企业经营成本。扎实开展“政银企”对接合作，努力缓解企业融资难融资贵问题。引导民营企业加快建立现代企业制度、完善治理结构，提高抗风险能力和市场竞争力。我们要全心营造一视同仁、公平竞争的发展环境，支持广大民营企业家心无旁骛创新创造，踏踏实实办好企业。推进军民融合深度发展。精准对接国家、省军民融合重大工程和产业投资基金，布局实施一批军民融合重点项目，加强军民融合创新示范基地、中关村联创军民融合产业园、中国航天环境实验室等载体建设，不断提高“军转民”“民参军”水平。全面提升质量品牌。开展质量提升攻坚行动，创建全国质量强市示范城市。质量之魂，存于匠心。要大力弘扬劳模精神、劳动精神、工匠精神，加快建设一支知识型、技能型、创新型劳动者大军，营造劳动光荣的社会风尚和精益求精的敬业风气。

（三）深度经略海洋，建设海洋经济大市。发挥我市海洋资源丰富、海洋经济基础强的优势，全力做好海洋开发这篇大文章。建设世界一流的海洋港口。推进全市港口资源整合，统筹建设10个港区，完成西港区30万吨级航道及拓宽工程，推进原油码头二期及LNG接收站等项目，积极开辟新航线，拓展大通关，延伸无水港，打造区域性航运中心。建设具有烟台特色的现代海洋产业体系。突破发展海洋渔业、海工装备制造、海洋生物医药、海洋文化旅游、海洋交通运输、海水淡化和综合利用“六大海洋产业”。高水平建设开发区“蓝色种业硅谷”，组建海洋牧场发展联盟，统筹建设四十里湾海域、庙岛群岛海域、莱州湾东部海域、烟台南部海域等4个海洋牧场示范区，启动10个海洋牧场示范项目，打造海洋牧场示范之城。谋划实施一批滨海工业园区海水淡化工程，加快海水利用产业化步伐。建设绿色可持续的海洋生态环境。严格落实生态红线管控措施和海洋生态补偿制度，全面推进湾长制、港长制，实施蓝色海湾治理、重要河口治理、海岸线整治修复等工程，有效防范各类海洋灾害风险。推进长岛海洋生态文明综合试验区全面发展，争创海洋类国家公园。

（四）扎实推进乡村振兴战略，高标准谱写齐鲁样板烟台篇章。始终坚持农业农村优先发展，聚焦“五个振兴”，深化农业供给侧结构性改革，巩固发展农业农村好形势。让产业更发达。紧紧围绕农民增收这个核心，加快构建现代乡村产业体系，推进农业由增产导向转向提质导向。实施质量兴农、品牌强农、绿色富农行动，培育壮大特色品牌，增加优质绿色农产品供给。紧抓粮食生产不放松，建成粮油核心示范区3万亩以上，确保粮食产能稳得住、发展可持续。推进涉农资金统筹整合，切实提高支农资金使用效益。高度重视农产品质量安全，新认证“三品一标”110个，整建制创建国家农产品质量安全示范市。毫不放松地抓好以非洲猪瘟为重点的重大动物疫病防控工作，确保畜禽产品质量安全和市场供应稳定。让人才更兴旺。把人力资本开发放在首要位置，实施返乡下乡创业培训专项行动和引才回乡工程，统筹抓好农业科技人才、农业专业人才、新型职业农民、农村乡土人才、农村创新创业人才5支乡村人才队伍建设，培训各类农业人才6万人次以上。让乡风更文明。深入推进移风易俗，加快建设新时代文明实践中心，持续提升文明村镇建设标准，整合盘活基层乡镇文化站和各类农村文化资源，培育文明乡风、良好家风、淳朴民风。让生态更宜居。扎实开展农村人居环境整治，基本完成农村无害化卫生厕所改造任务，加强垃圾污水处理设施建设，改善村容村貌，打造农民安居乐业的美丽家园。推进“四好农村路”建设，改造农村公路，整治村级公路隐患路段。推进城乡公交一体化，建设客运站点1000个。全力解决30万农村群众饮水安全问题。坚决落实最严格的耕地保护制度和节约集约用地制度，不折不扣开展“大棚房”问题专项清理，对占用耕地违法违规建设非农设施行为坚决予以整治。让组织更有力。加强农村党的基层组织建设，深入推进“三创两提升”工程，加大党支部领办合作社力度，争取建成示范型合作社1200家。完成镇街、村居党群服务中心规范化建设。建立健全党组织领导的自治、法治、德治相结合的乡村治理体系，打造充满活力、和谐有序的善治乡村。

（五）继续打好三大攻坚战，牢牢守住发展底线。严密防控重大风险。高度重视化解企业风险，强化企业流动性等重点领域金融风险监测预警，继续协助金融机构处置不良资产，严厉打击逃废银行债务行为，坚决遏制各类非法集资活动。加强政府债务管理，严防新增各类政府隐性债务，有序化解政府存量债务，坚决守住不发生系统性金融风险底线。着力巩固提升脱贫攻坚成果。去年我市基本完成脱贫攻坚任务，今年要把工作的着力点放到巩固提升脱贫质量上，持续强化“两不愁三保障”，减少和防止贫困人口返贫，探索收入水平略高于建档立卡贫困户的群体政策支持机制，确保全面建成小康路上一户不落、一人不少。强力推进污染防治。深入贯彻落实习近平生态文明思想，以更高的站位、更严的标准、更大的力度推进生态环保工作，以钉钉子精神把生态文明建设推向深入。坚定不移抓好中央、省生态环保督察反馈意见和“回头看”移交问题整改，深入实施“四减四增”三年行动，综合治理燃煤、扬尘、柴油车、挥发性有机物污染，持续开展“散乱污”企业综合整治，市区空气质量稳定达到国家二级标准并持续改善。实施最严格水资源管理制度，完成170个县级饮用水源地保护区环境问题清理整治。坚决打好渤海综合治理攻坚战，坚决守住环渤海生态保护红线，坚决杜绝各类违规填海行为。加强昆嵛山自然保护区生态建设，抓好各类自然保护区生态修复，持续改善生态环境。大力实施全域绿化三年行动计划，完成造林和生态修复13万亩、森林抚育20万亩、湿地保护与恢复8.5万亩。统筹山水林田湖草系统治理，让良好的生态环境成为展现烟台形象的靓丽名片，成为全市人民的永久福利！

（六）深化关键领域改革，进一步增强发展活力。紧紧盯住痛点、难点、堵点，完善体制机制，推动各项改革向深处走、向实处做。深化“放管服”改革。重点围绕企业开办、不动产登记、施工许可等10个方面开展提升行动，努力提高群众和企业办事效率，全力打造“审批事项少、办事效率高、服务质量优、群众获得感强”的营商环境。深化国资国企改革。纵深推进行政事业单位国有资产运营管理改革，强化市属国有企业混合所有制改革，积极引进战略投资者，鼓励职工持股参与改革，启动国有企业工资决定机制改革，做强做优做大国有资本。深化财税、金融和投融资改革。加快推进市以下财政事权与支出责任划分改革，建立科学规范的预算绩效管理体系。进一步优化国有资本布局，促进蓝天、城发、财金等市级投融资公司发展壮大，鼓励民营资本参与全市公共基础设施建设，进一步放开市场、增添活力。丰富和发展地方金融组织业态，提高金融体系服务实体经济能力。加快建设区域性基金管理中心。深化农村改革。推进农村土地制度改革，加快农村集体产权制度改革，普遍成立股份经济合作社等新型农村经济组织。深化医药卫生体制改革。统筹实施医疗医保医药“三医”联动，建成10个医共体，继续开展居民长期护理保险试点，把更多救命救急的好药纳入医保，进一步方便群众看病就医。加快推进教育、文化、养老服务、综合行政执法等其他改革事项。

（七）推动全方位对外开放，打造开放发展新高地。强力推进“双招双引”。深化招商体制改革和招商模式创新，更加重视专业招商，提高招商的精准度。以日韩为重点，全力加大招商引资和产业合作力度，进一步扩大开放优势。全年力争引进到位市外资金1200亿元、“三类500强”投资项目80个以上。全方位开展招才引智，实施重点企业（园区）引才计划，建立优秀人才来烟就业创业生活补贴制度，举办海内外精英创业大赛、高端人才烟台行、桑梓创客计划等活动，推进八角湾国际人才港、烟台高层次人才创业园建设，打造“慧聚烟台”招才品牌，新增高层次人才5000名以上。加强与国际友城和海外侨社组织的交流合作，重视发展烟台在各地的商会。全面拓展对外贸易。加强与“一带一路”沿线国家和地区的经贸往来，推动更多企业参与利用集装箱远洋航线和欧亚班列组货运营。优化口岸营商环境，提高出口退税效率，促进机电产品和服务贸易出口，扩大先进技术和关键零部件进口。建设跨境电商公共服务平台和境外商品展示中心，争创中国服务外包示范城市。推动园区集约化特色化发展。烟台经济技术开发区借助“两港一站”配套优势，大力发展先进制造业和高端服务业，建设全市现代化经济中心。烟台高新区发挥国家自主创新示范区先行先试优势，统筹抓好金山湾区片建设，加快聚集高层次人才、高端研发机构和高新技术产业，打造创新创业新高地。烟台保税港区抓住山东自贸试验区申建契机，搞好东区发展和西迁置换，争取更多自贸区创新制度复制推广。招远开发区打造先进制造业、高新技术产业和生产性服务业集聚发展新平台。高标准建设中韩（烟台）产业园，规划建设中日、中德、中意等国别特色园区。统筹推进各省级开发区差异化协同发展，打造区域高质量发展龙头。进一步繁荣会展经济。加快会展场馆建设，精心策划和办好国际果蔬食品博览会、国际葡萄酒博览会、国际工业设计产业博览会、医药创新与发展国际会议等一批特色展会，争取举办有规模、有影响力的展会活动100个以上。

（八）完善提升城市功能，建设宜业宜居宜游城市。增强基础设施保障能力。争取环渤海潍烟高铁和蓬莱国际机场二期工程开工建设，加快大莱龙铁路扩能改造。推进文莱高速公路，实施7个国省干线公路项目。做好城市轨道交通前期工作。推动海阳核电3、4号机组开工建设，力争清洁能源装机容量达到650万千瓦。继续实施全域治水三年攻坚行动，推进河库水系连通、小型水库除险加固等66项工程，年增加供水能力1.1亿立方米。加快老岚水库前期工作，力争2020年开工建设，2024年具备蓄水条件。高质量组织城市重点项目建设。抓好新一轮城市总体规划编制。实施好83项市区城建重点项目，完成年度投资253.5亿元。稳步推进以朝阳、所城为主体的烟台古城建设，打造守护烟台历史文脉、承继烟台地域遗产的旅游新地标、文化记忆场。启动烟台海上世界项目，建设芝罘湾国际邮轮母港，打造集“船港城游购娱”于一体的高端商旅综合体。建成启用城发城市广场、市民公园和市档案馆。推进幸福北部新城开发，打造新的中心城市功能区。组织开展金沙江路等22条城市道路工程建设，塔山南路部分隧道贯通，建成滨海路慢行系统。开工建设棚改安置房3607套，改造老旧小区13个，改造背街小巷21条。推进房地产市场平稳健康发展，坚决打击各类投机炒房行为，完善住房市场体系和保障体系，为8000户城镇住房困难家庭发放租赁补贴。加强供水、供电、供气、供热等配套基础设施建设，建设海绵城市20平方公里，实施一批垃圾焚烧发电、污水处理厂、地下管网建设工程。精心精致管理城市。大力开展“拆违、治堵、增亮、添绿”行动，重拳出击拆除违法建设，全面完成城市绿线、城市出入口及侵占山体山林等重点违法建设拆除任务。全力治理交通拥堵，加强市区交通设施建设，推进红旗路快速化改造，打通城市断头路，提升公交服务水平，实行市区公交“一元一票制”，让市民出行更便捷。抓好滨海景观带等重点区域亮化，提升城市绿化景观，新建改造一批社区公园、街头游园，丰富城市绿化纹理，让广大群众生活在绿意盎然的美好环境之中。

（九）积极发展社会事业，切实保障和改善民生。我们要更好地顺应人民群众对美好生活的向往，集中办好100项惠民实事，确保发展前进一步、民生改善就跟进一步，让全市人民享有更直接的获得感、幸福感和安全感。提高就业质量。高度重视稳就业工作，及时掌握重点行业用工变化，把扩大就业的立足点更多地放在提高人力资源素质上，吸引更多有知识、有技能的年轻人来烟发展，全年新增城镇就业13万人，城镇登记失业率控制在3.5%以内。办好人民满意的教育。推动教育资源优质均衡发展，继续做好消除大班额和高中阶段教育普及攻坚。加大学前教育投入，增加公办幼儿园数量，新投用2.7万个幼儿学位。加快发展高等教育，高起点规划建设金山湾科教园区。高度重视职业教育和特殊教育，鼓励社会力量办学，推动教育国际合作。完善社会保障体系。深入实施全民参保计划，稳步提高养老、失业、工伤等待遇水平。持续提升城乡低保标准，保障困难群众基本生活。加强退役军人服务保障体系建设，做好退役军人优抚安置等工作。发展慈善事业和残疾人事业，维护妇女合法权益，做好农村留守儿童关爱保护和困境儿童保障工作。建设国家健康城市。加快建设烟台山医院新院、国际肿瘤医学中心，创建10个省级卫生乡镇，重视中医药传承发展。加强医养结合试点市建设，抓好14个养老项目建设，新增10处医养结合服务机构、3000张医养结合床位。完善城市社区服务功能，推进社区养老服务中心建设，大力举办社区食堂，更加便捷地服务广大市民。统筹发展群众体育、竞技体育和体育产业，更新一批全民健身设施，建成烟台射击射箭竞技体育运动学校，举办市第九届全民健身运动会。坚持文化惠民。积极培育和践行社会主义核心价值观，推动优秀传统文化创造性转化、创新性发展，推进胶东（烟台）党性教育基地建设。繁荣发展党报党刊和广播电视、新闻出版事业。做好庆祝新中国成立70周年系列文化活动，提升城市文化品位。建设烟台图书馆新馆，促进县级图书馆提档升级，持续开展全民阅读活动。加强文化遗产保护。建设平安法治烟台。建立立体化社会治安防控体系和多元化矛盾纠纷排查化解体系，加强和规范社区网格化服务管理工作。推进公安基层基础攻坚，深入开展扫黑除恶专项斗争和三年禁毒人民战争，严厉打击各类违法犯罪行为。大力实施“七五”普法，完善公共法律服务体系，创建法治建设示范城市。加强和改进信访工作，加大力度化解信访积案。严格落实安全生产责任制，强化重点领域监管，构建城市公共安全体系，坚决防范和遏制重特大事故发生。加强应急管理，强化应急救援力量建设，提升综合应急救援能力。加强国防教育、国防动员、双拥共建、民兵预备役、人民防空等工作，建成市国防教育训练基地保障中心。

三、建设人民满意的服务型政府

新的一年，政府系统要切实加强自身建设，着力提高治理体系和治理能力现代化，全面增强公信力和执行力，努力为人民服务、对人民负责、受人民监督、让人民满意。

（一）旗帜鲜明讲政治。自觉用习近平新时代中国特色社会主义思想武装头脑、指导实践、推动工作，着力在学懂弄通做实上下功夫，树牢“四个意识”，坚定“四个自信”，做到“两个维护”，始终在思想上政治上行动上同以习近平同志为核心的党中央保持高度一致，不折不扣把党中央国务院、省委省政府和市委的决策部署落地落实，确保政令畅通、令行禁止。坚持把抓好党建作为最大政绩，从严从实加强市政府党组和政府系统党的建设，落实意识形态工作责任制，确保政府系统政治合格、全面过硬。

（二）依法行政重法治。加强法治政府建设，严格遵守宪法法律，自觉运用法治思维和法治方式深化改革、推动发展、化解矛盾，做到法定职责必须为、法无授权不可为，推动政府工作全面进入法治轨道。认真执行市人大及其常委会的决议决定，主动加强同人民政协的民主协商，自觉接受市人大及其常委会监督、政协民主监督、社会舆论监督，高质量办好人大代表建议和政协提案。扎实做好地方立法工作。深入推进政务公开，及时回应群众关切。健全完善社会信用体系，提升全社会诚信水平，努力建设权责法定、公正严明、守法诚信的法治政府。

（三）牢记宗旨服好务。自觉践行以人民为中心的发展思想，始终把群众的冷暖放在心上，注意倾听群众的呼声和意见，既要谋划实施好普惠性的民生大事，更要用心解决好群众的身边小事。建设迅捷高效的12345政务服务热线，及时解决群众反映的问题。着力构建亲清新型政商关系，各级政府都要理解企业家、尊重企业家，不断优化服务、提高效能，做到有求必应、无需不扰，千方百计为企业排忧解难，让各类市场主体在阳光雨露下茁壮成长。

（四）改进作风有担当。任何成绩都是拼出来干出来的。各级政府及工作人员要奋发有为、干字当头，深入开展“思想大解放、作风大改进、工作大落实”，提升标杆、勇于担当、争创一流，把各项工作一抓到底，干就干成一样，做就做好一件。进一步完善激励约束、容错纠错机制，严肃问责不作为、慢作为、乱作为的干部，旗帜鲜明为敢于担当者担当、为敢于负责者负责，锻造一支真抓实干、不务虚功、主动担责、全力尽责的公务员队伍。

（五）从严治政守纪律。始终把纪律和规矩挺在前面，严格落实全面从严治党主体责任，全面加强党风廉政建设，坚决贯彻落实中央八项规定精神，驰而不息纠正“四风”，特别是力戒形式主义、官僚主义。加强审计监督，完善统计体制，坚决杜绝一切形象工程、政绩工程，反对任何数字造假。自觉接受纪检监察监督，以零容忍态度严厉惩治腐败，干干净净为人民做事，营造风清气正的政治生态。

各位代表，新时代是奋斗者的时代，奋斗者的脚步永不停歇！让我们更加紧密地团结在以习近平同志为核心的党中央周围，在省委、省政府和市委的坚强领导下，不忘初心、牢记使命，一张蓝图绘到底，脚踏实地、锲而不舍、只争朝夕，加快建设制造业强市、海洋经济大市、宜业宜居宜游城市和现代化国际滨海城市，以优异成绩迎接中华人民共和国成立70周年！
